# Expanding spectrum, intrafamilial diversity, and therapeutic challenges from 15 patients with heterozygous CARD11-associated diseases: A single center experience

**DOI:** 10.3389/fimmu.2022.1020927

**Published:** 2022-11-03

**Authors:** Luciano Urdinez, Lorenzo Erra, Alejandro M. Palma, María F. Mercogliano, Julieta Belén Fernandez, Emma Prieto, Verónica Goris, Andrea Bernasconi, Marianela Sanz, Mariana Villa, Carolina Bouso, Lucia Caputi, Belen Quesada, Daniel Solis, Anabel Aguirre Bruzzo, Maria Martha Katsicas, Laura Galluzzo, Christian Weyersberg, Marcela Bocian, Maria Marta Bujan, Matías Oleastro, María B. Almejun, Silvia Danielian

**Affiliations:** ^1^ Servicio de Inmunología y Reumatología, Hospital Nacional de Pediatría Juan P. Garrahan, Buenos Aires, Argentina; ^2^ Laboratorio de Biofisicoquímica de Proteínas, Departamento de Química Biológica, Instituto de Quimica Biologica de Facultad de Ciencias Biologicas y Naturales (IQUIBICEN), Facultad de Ciencias Exactas y Naturales, Universidad de Buenos Aires, Buenos Aires, Argentina; ^3^ Laboratorio de Genética en Endocrinología, Instituto de Biociencias, Biotecnologia y Biologia Translacional (IB3), Departamento de Fisiología, Biología Molecular y Celular, Facultad de Ciencias Exactas y Naturales, Universidad de Buenos Aires, Buenos Aires, Argentina; ^4^ Servicio de Anatomía Patológica, Hospital Nacional de Pediatría Juan P. Garrahan, Buenos Aires, Argentina; ^5^ Servicio de Gastroenterología, Hospital Nacional de Pediatría Juan P. Garrahan, Buenos Aires, Argentina; ^6^ Servicio de Dermatología, Hospital Nacional de Pediatría Juan P. Garrahan, Buenos Aires, Argentina

**Keywords:** CARD11, inborn error of immunity, atopic, lymphoproliferation, gain of function, dominant negative, benta, cadins

## Abstract

CARD11-associated diseases are monogenic inborn errors of immunity involving immunodeficiency, predisposition to malignancy and immune dysregulation such as lymphoproliferation, inflammation, atopic and autoimmune manifestations. Defects in *CARD11* can present as mutations that confer a complete or a partial loss of function (LOF) or contrarily, a gain of function (GOF) of the affected gene product. We report clinical characteristics, immunophenotypes and genotypes of 15 patients from our center presenting with CARD11-associated diseases. Index cases are pediatric patients followed in our immunology division who had access to next generation sequencing studies. Variant significance was defined by functional analysis in cultured cells transfected with a wild type and/or with mutated h*CARD11* constructs. Cytoplasmic aggregation of *CARD11* products was evaluated by immunofluorescence. Nine index patients with 9 unique heterozygous *CARD11* variants were identified. At the time of the identification, 7 variants previously unreported required functional validation. Altogether, four variants showed a GOF effect as well a spontaneous aggregation in the cytoplasm, leading to B cell expansion with NF-κB and T cell anergy (BENTA) diagnosis. Additional four variants showing a LOF activity were considered as causative of CARD11-associated atopy with dominant interference of NF-kB signaling (CADINS). The remaining variant exhibited a neutral functional assay excluding its carrier from further analysis. Family segregation studies expanded to 15 individuals the number of patients presenting CARD11-associated disease. A thorough clinical, immunophenotypical, and therapeutic management evaluation was performed on these patients (5 BENTA and 10 CADINS). A remarkable variability of disease expression was clearly noted among BENTA as well as in CADINS patients, even within multiplex families. Identification of novel *CARD11* variants required functional studies to validate their pathogenic activity. In our cohort BENTA phenotype exhibited a more severe and expanded clinical spectrum than previously reported, e.g., severe hematological and extra hematological autoimmunity and 3 fatal outcomes. The growing number of patients with dysmorphic facial features strengthen the inclusion of extra-immune characteristics as part of the CADINS spectrum. CARD11-associated diseases represent a challenging group of disorders from the diagnostic and therapeutic standpoint, especially BENTA cases that can undergo a more severe progression than previously described.

## 1 Introduction

Inborn errors of immunity (IEI) comprise over 450 entities caused by monogenic defects that involve immunodeficiency, allergy, predisposition to malignancy and immune dysregulation such as lymphoproliferation, autoinflammation, atopic and autoimmune manifestations (1). Defining the genetic and molecular basis of these diseases has enhanced our understanding about the development and function of the immune system. Advances in the use of Next Generation Sequencing (NGS) technologies have made it possible to identify an expanding number of variants in a particular gene along with increasingly broad phenotypes (2). Some of these genetic defects can present as variants that confer loss of function (LOF) or contrarily, a gain of function (GOF) of the affected gene product. One gene presenting with variants leading to increased or decreased function of the coding protein is Caspase Activation and Recruitment Domain 11 (*CARD11*, also known as *CARMA1*) *(* 3).

CARD11 encodes a lymphocyte-specific scaffold protein assembling in a complex with B-cell chronic lymphocytic leukemia/lymphoma 10 (BCL10) and mucosa-associated lymphoid tissue lymphoma translocation gene 1 (MALT1) [CBM complex]. This signaling complex is a critical adaptor that mediates immune responses downstream of both cell surface and intracellular receptors, including the B cell receptor (BCR) and T cell receptor (TCR) (4, 5). This scaffold signaling complex is necessary inter alia for proper nuclear factor kappa B (NF-κB) (6), c-Jun N-terminal kinase (JNK) (7), and mechanistic target of rapamycin complex (mTORC1) (8) activation, as well as for regulating lymphocyte activation, proliferation, and survival (4, 5, 9, 10).

In the last years, germline pathogenic variants in CARD11 have been linked to various distinct IEI (3), collectively referred in this paper as CARD11-associated diseases. Given that CARD11 must be oligomerized to continue the downstream signaling cascade, heterozygous variants may increase or decrease protein function (4). Germline GOF variants cause a lymphoproliferative disorder called B cell expansion with NF-κB and T cell anergy (BENTA) (11). Although the clinical phenotype was first described in 1971 (12), the genetic identification that BENTA was caused by germline CARD11 GOF variants was done in 2012 (13). Interestingly, many pathogenic variants found in BENTA patients are also found as somatic mutation in patients with various lymphoid malignancies (14–16).

Heterozygous LOF dominant negative (DN) variants in CARD11 cause CARD11-associated atopy with dominant interference of NF-κB signaling (CADINS), a combined immunodeficiency with severe atopic disease and other variable immunodeficiency phenotypes (17–19). pathogenic features of both GOF and LOF patients were also reported (20). Besides, biallelic null variants in CARD11 lead to a profound combined immunodeficiency (21, 22).

Since BENTA and CADINS descriptions, efforts have been made to properly characterize the clinical and immunological spectrum of these diseases to enable optimal treatment strategies and outcomes. So far, the biggest multicenter cohort of CADINS was reported by Dorjbal et al. with 60 affected individuals (23). In contrast, less than 20 BENTA patients have been reported in several publications until today (24–30).

Functional impact of novel and very rare CARD11 variants identified by NGS technologies, such as gene panel or whole-exome sequencing (WES) in patients under immune evaluation, cannot be predicted with absolute certainty. Therefore, functional assessment of new identified variants must be used for a proper interpretation (23). Indeed, both follow-up and treatment markedly diverge for the different phenotypes described in patients carrying pathogenic variants in CARD11.

Here, we present a comprehensive clinical, immunological, and genetic data, with functional characterization of CARD11 variants in 15 individuals, 10 CADINS [including 3 related patients previously reported in Ma et al. (17)] and 5 BENTA patients, from our clinical care center.

## 2 Materials and methods

### 2.1 Patients

**Figure 1:**
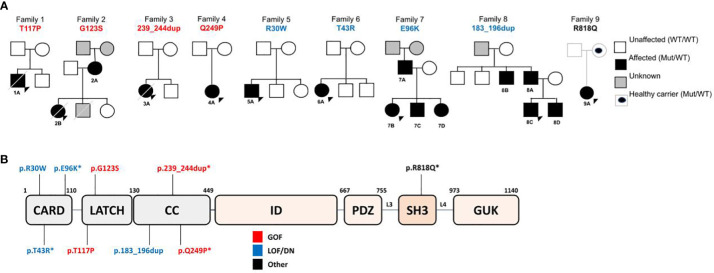
**(A)** Family Pedigrees, with CARD11 variants found and affected family members. **(B)** Schematic representation of CARD11 protein, with variants found in our cohort.

Informed written consent was obtained from all participating patients and their family members according to protocols approved by the internal ethics review board of the Hospital Garrahan. For clinical and phenotyping data see Results and **Supplementary Materials**. See complete pedigrees in **Figure 1A**.

### 2.2 Molecular studies

Next Generation Sequencing studies were performed by a Custom IEI gene panel and WES (whole exome sequencing). Custom IEI gene panel contains 154 genes (**Supplementary Figure 1** manufactured by Agilent SureSelect Technologies, for the exon capture the SureSelect XT HS2 DNA Reagent Kit was used according to manufacturer’s instructions. Sequencing was performed in an Illumina MiSeqTM System as 2 × 300 bp read lengths and 200X average depth. DNA reads were mapped to the GRCh38 version of the human reference genome.

### 2.3 Variant prioritization

Technical duplicates were removed using Piccard and “HaplotypeCaller” algorithm (GATK) (31, 32)was used to call SNPs and indels.

Variant filtering and prioritization was realized using BPlatform (https://www.bitgenia.com/b-platform/) according to minor allele frequency by using dbSNP (Single Nucleotide Polymorphism database, July 15, 2019) (33), 1000 Genomes (https://www.internationalgenome.org/, March 10, 2019), dbNSFP (Non-synonymous single-nucleotide variants database, March 20, 2019) (34), ExAC (Exome Aggregation Consortium, Versión 1.0, July 10, 2019) (35)and ClinVar (https://www.ncbi.nlm.nih.gov/clinvar/, March 22, 2019). The potential impact of the amino acid substitution in the protein was assessed with PolyPhen2 (Polymorphism Phenotyping, October 10, 2019) (36), the amino acid conservation was examined with Clustal Omega (37) (http://www.clustal.org/), Mutation Taster (38) (http://www.mutationtaster.org/) and for scoring the deleteriousness of single nucleotides variants as well as deletion/insertion variants Combined Annotation Dependent Depletion (CADD) tool (https://cadd.gs.washington.edu).

### 2.4 Sanger sequencing

*CARD11* Sanger sequencing was used to confirm NGS-detected variants, to screen family members and to corroborate the pUNO-hCARD11-FLAG mutagenesis. gDNA was PCR-amplified using GoTaq polymerase (Promega) and exon specific primers. Amplicons were sequenced in both directions using the Big Dye Terminator version 1.1 cycle sequencing kit and an Applied Biosystems 3130xl Genetic Analyzer (Life Technologies).

### 2.5 Plasmid DNA cloning

Site-directed mutagenesis was used to introduce single point nucleotide variants into wild type (WT) pUNO-hCARD11-FLAG. To generate the mutated forms the QuickChange II site-directed mutagenesis kit (Stratagene; Agilent, Santa Clara, CA) was used following the manufacturer’s instructions. QuickChange XL II primer design software (Agilent) was used for specific primer designs. Briefly, the mutagenesis reaction was performed with PfuI polymerase (Roche, Mannheim, Germany) in a ThermoFisher Scientific thermocycler with the following conditions: 94°C for 5 minutes, cycled 25 times at 94°C for 15 sec, 58°C for 1 min, 68°C for 8 minutes, followed by DpnI digestion of methylated DNA template (Thermo Fisher). All plasmids were purified using QIAGEN Mini Prep kit with the Mira-Prep protocol (39) from transformed competent DH5α E. coli (New EnglandBioLabs) selected with blastidicin S (GoldBio).

### 2.6 Cell transfection assays

Jurkat T-cell line obtained from ATCC (clone E6.1) and CARD11-deficient Jurkat T-cells (JPM50.6) expressing a NF-κB-driven GFP reporter, kindly provided by Andrew Snow (13), were cultured in RPMI 1640-GlutaMAX 1X (Gibco, Brazil) supplemented with 10% FCS (Gibco), 1% HEPES 1M (Sigma); 1% sodium pyruvate 100 X (Gibco); 1% of penicillin and streptomycin 100X (Gibco). HEK293T cell line was cultured with DMEM high glucose (Gibco) pH (7.4) with 3.7g of sodium bicarbonate for 1L, supplemented with 10% FCS (Gibco), 1% of penicillin and streptomycin 100X (Gibco).

JPM50.6 and Jurkat T-cells were cultured and transfected as previously described (17). Briefly 2.5 × 10^6^ cells/cuvette (BIO-RAD) were electroporated with 2 μg pUNO-hCARD11-FLAG (WT and variants) in 0.4 mL RPMI 10% FBS (with no antibiotics) using Gene PulserXcell II (BIO-RAD) at 260 V, 950 μF. For luciferase assays, Jurkat T-cells were also electroporated with 2 μg de pNF-κB-luc and 300 mg Renilla (NF-κB activation was calculated with luciferase/renilla ratio). Half of these cells were stimulated 24h after transfection with 1 μg/ml of anti-CD3 OKT3 + anti-CD28 (BioLegend). NF-κB activity was measured 24h later *via* detection of κB-GFP reporter expression in JPM50.6 cells using FACS ARIA (Becton Dickinson) and quantified based on mean fluorescence intensity (MFI), or *via* Dual-Luciferase Reporter Assay (Promega) in Jurkat T-cells using a Microplate Reader. Relative NF-κB activation in Jurkat T cells transfectants was calculated by normalizing the relative ratio of firefly to Renilla luciferase signals.

HEK293T cells were plated at 1,5x10^4^ cells/well in a 96-well plate and transfected using Lipofectamine 3000 (Invitrogen) with 45 ng pNF-κB-luc and pUNO-hCARD11-FLAG (WT, mutants and variants) and 6 ng pRenilla. NF-κB activity was quantified 48h after transfection by Dual-Luciferase Reporter Assay (Promega) using Marca Microplate Reader.

### 2.7 Immunoblotting

Whole cell lysates for each of the variant’s transfection were prepared in RIPA lysis buffer with 1 mM PMSF and protease inhibitors (ActiveMotifs) as indicated by the manufacturer. Then lysates were centrifuged at 12000 g for 15 minutes at 4°C and protein concentration was quantified by Bradford protein assay (40). To verify CARD11 expression, lysates (25 ug) were solubilized in sample buffer and then were run on 8% Tris-GlycineSDS gels (Bio-Rad) and transferred to nitrocellulose membrane 0.2 μm (Bio-Rad). Membranes were then blocked and incubated with the following antibodies: anti-FLAG M2 (anti-DYKDDDDK) (F3165) (Sigma); anti-β-actin (8H10D10) (Cell Signaling Technologies). Primary antibodies were detected using HRP-conjugated secondary antibodies (Cell Signaling Technologies) and detected with ECL Advance Western Blotting Detection Kit (BIO-RAD) according to the manufacturer’s indications. Images were obtained with G:Box (Syngene, Bangalore, India) and analyzed with ImageJ software (NIH).

### 2.8 Immunofluorescence

HEK293T cells were grown in round glass coverslips and post transfection with the indicated plasmids, the cells were fixed and permeabilized with ice-cold methanol and then washed. The non-specific binding sites were blocked with BSA 1% PBS for 30 min. Then, cells were incubated ON at 4°C with anti-FLAG. Secondary Alexa Fluor 488-conjugated antibody (#4414, Cell Signaling Technologies) was incubated for 1h at room temperature. Negative controls were carried out using PBS 1% BSA instead of the primary antibody. Nuclei were stained with DAPI 2 µg/ml (Sigma Aldrich, Saint Louis, MO). Finally, the coverslips were mounted on glass slides with mounting medium (Prolong Gold Antifade Reagent, ThermoFisher). Cells were analyzed using an Olympus DSU IX83 Spinning Disk microscope.

### 2.9 Statistical analysis

For JPM50.6 cell transfections, 2-way ANOVAs with Sidak’s correction was used to compare GFP-MFI between WT and CARD11 mutants or variants. For Jurkat and HEK293T cell transfections, Wilcoxon matched-pairs signed rank tests were used to compare luciferase activation between WT and CARD11 mutants. Statistics data were analyzed using GraphPad Prism 7 software (GraphPad Software Inc.).

### 2.10 Pathology

Tissue was fixed in formalin 10% and paraffin embedded. Four-micron cuts were colored with hematoxilin-eosin. CD20, CD3, Ki67, CD30 and other immunostains were performed when necessary.

## 3 Results

Since 2016, NGS technologies are regularly available in our center. A total of 214 patients with immunodeficiency, atopic and/or dysregulatory phenotypes followed in our Immunology and Rheumatology division were studied through a Custom IEI gene panel (Agilent Technologies, see **Supplemental Figure 1** for gene list) or an external WES. A total of 9 patients from 9 unrelated families (**Figure 1A**) were found to display 9 different novel or very rare heterozygous germline *CARD11* variants. No patient harbored biallelic variants in our cohort.

### 3.1 Variant *in silico* analysis

All the *CARD11* variants identified in families from our cohort (**Figure 1B**) are absent from gnomAD v3.1.2 and ExAC v.1 except for p.Arg818Gln showing a very low population frequency (4.6 x 10-5 and 6.6 x 10-5, respectively). We used Clustal Omega (37) and Mutation Taster Phast Cons (http://www.mutationtaster.org/) to study the amino acid sequence alignment and conservation between species. From both analyses the novel variants presented a high degree of amino acid conservation in homologous proteins found even in species evolutionarily distant as Danio rerio (Zebrafish) or Xenopus (**Supplementary Figure 2**). The PolyPhen2 (Polymorphism phenotyping) (41)values are close to 1 for all the novel missense mutations meaning that the variants are predicted to be damaging by altering the local structure of the protein (data not shown). There is a general agreement that https://cadd.gs.washington.edu/Combined Annotation Dependent Depletion (CADD) results a better tool for scoring the deleteriousness of single nucleotide variants as well as insertion/deletions variants in *CARD11*. Indeed, CADD scores for all variants identified in this cohort are higher than 22, threshold above which *CARD11* changes are likely pathogenic (**Supplementary Figure 2**).

### 3.2 Four of the novel or rare CARD11 variants affected NF-κB activation in T-cell lines

Nine unique variants were identified (**Figure 1B**) through custom IEI panel or external WES (p.Arg30Trp, p.Thr43Arg, p.Glu96Lys, p.Thr117Pro, p.Gly123Ser, p.183_196dup, p.239_244dup, p.Gln249Pro, p.Arg818Gln). The previously reported pathogenic mutations from those identified in our cohort were p.Arg30Trp (LOF/DN) (18)and p.Gly123Ser (GOF) (13, 42). Regarding p.183_196dup variant found in family 8, functional studies showing a LOF/DN effect were the subject of collaboration with an external group and reported in Ma et al., 2017 (17). Likewise, for p.239_244dup variant an increased NF-κB activation has been demonstrated elsewhere (Andrew L. Snow, personal communication).

**Figure 2:**
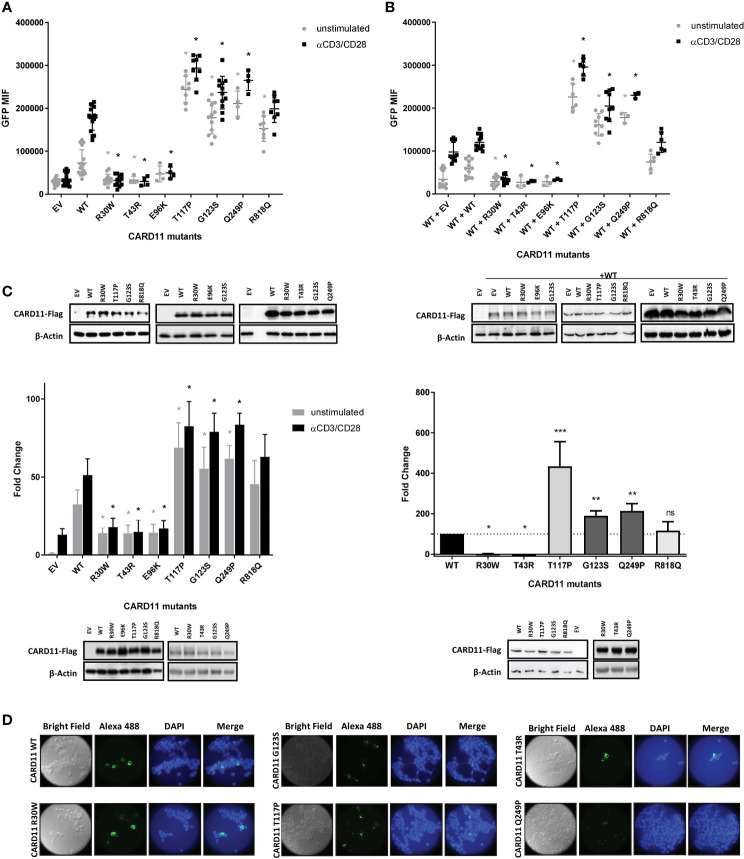
Reporter gene assays of CARD11 variants in JPM50.6,Jurkat T cells and HEK293T cell lines. **(A)** Quantification of Flow cytometric NF-κB-driven GFP reporter expression in JPM50.6 cells transfected with empty vector (EV), WT or mutant CARD11 constructs, unstimulated or stimulated with anti-CD3 and anti-CD28 for 24 h. (mean fluorescence intensity (MFI) of GFP+ cells). Data are mean ± s.e.m. (EV and WT n=16; p.R30W and p.G123S n=12, p.T117P and p.R818Q n=8; p.T43R, p.E96K and p.Q249P n=4) Two-way ANOVA with Sidak’s multiple t-test. *p<0.05 for each mutant versus WT with or without stimulation (EV<0.0001, <0.0001; p.R30W p=0.0110, <0.0001; p.T43R p=0.0485, <0.0001; p.E96K p=0.4627, <0.0001; p.T117P p<0.0001, <0.0001; p.G123S p<0.0001, <0.0001; p.Q249P <0.0001, <0.0001; p.R818Q p<0.0001, p=0.3119). **(B)** Quantification of Flow cytometric analysis (GFP MFI) of JPM50.6 cells transfected with WT CARD11 plus WT or mutant constructs and stimulated as in **(A)**. Data are mean ± s.e.m. (EV and WT n=12; p.R30W and p.G123S n=9, p.T117P and p.R818Q n= 6; p.T43R, p.E96K and p.Q249P n=3). Two-way ANOVA with Sidak’s multiple t test. *p<0.05 for each mutant versus WT with or without stimulation (EV p=0.0255, 0.0864; p.R30W p=0.0097, <0.0001; p.T43R p=0.1358, <0.0001; p.E96K p=0.1760, <0.0001; p.T117P p<0.0001, <0.0001; p.G123S p<0.0001, <0.0001; p.Q249P p<0.0001, <0.0001; p.R818Q p=0.7581, p>0.9999). **(C)** Left: Quantification of relative specific activity of Luciferase assay in transfected Jurkat T cells with empty vector (EV), WT or mutant CARD11 and reporter luciferase constructs, and stimulated as in **(A)**. Data represent fold change in luciferase activity normalized to renilla activity. Data are mean ± s.e.m. (EV, WT, p.R30W andp.G123S n=12; p.T117P andp.R818Q n=6; p.T43R, p.E96K and p.Q249P n=3). Two-way ANOVA with Sidak’s multiple t test. *p<0.05 for each mutant versus WT with or without stimulation (EV p<0.0001, <0.0001; p.R30W <0.0001, <0.0001; p.T43R p=0.0333, <0.0001; p.E96K p=0.0384, <0.0001; p.T117P p<0.0001, <0.0001; p.G123S <0.0001, <0.0001; p.Q249P <0.0001, <0.0001; p.R818Qp=0.0712, p=0.1365). Rigth: Quantification of relative specific activity of Luciferase assay in transfected HEK293T cells with empty vector (EV), WT or mutant CARD11 and reporter luciferase constructs. Data represent fold change in luciferase activity normalized to renilla activity. Data are mean ± s.e.m. Wilcoxon signed rank test *p<0.05, **p<0.01, ***p<0.005. **(A–C)** Immunoblot for CARD11-Flag expression in transfected cell lysates are at the bottom of each graphic β-Actin serves as a loading control. **(D)** HEK293T transfected with WT and CARD11 mutants constructs were analysed by confocal microscopy using anti-FLAG and Alexa-Fluor488-conjugated secondary Abs, nuclei were stained with DAPI. ns, not statistically significant.

Therefore, in this work we evaluated the effect of the remaining 5 novel or very rare CARD11 variants (p.Thr43Arg; p.Glu96Lys; p.Thr117Pro; p.Gln249Pro and p.Arg818Gln) by assessing the ability to alter TCR signaling. For this purpose, we transfected WT and mutant CARD11 expression constructs into JPM50.6 cell lines expressing a NF-κB-driven GFP reporter (43). In addition to these variants, we studied as controls the previously reported mutations we found in patient 1A and 2B: p.Arg30Trp and p.Gly123Ser, classified as LOF/DN and GOF, respectively (13, 18). As expected, stimulation with anti-CD3/CD28 for 24h in JPM50.6 cells transfected with pUNO-hCARD11-FLAG WT resulted in a significant increase in NF-κB activation (**Figure 2A**). As previously reported, the GOF mutant p.Gly123Ser induced constitutive GFP expression in the absence of stimulation (13). As well, the mutant reported as LOF/DN, p.Arg30Trp, failed to activate NF-κB after stimulation with anti-CD3/CD28 (**Figure 2A**).

Compared to WT, the novel variants pThr43Arg and p.Glu96Lys barely induced NF-κB activation upon stimulation with anti-CD3/CD28, suggesting a LOF impact (**Figure 2A**). Additionally, the ability of WT CARD11 to activate NF-κB pathway upon stimulation of the TCR was disrupted by co-transfection with either p.Thr43Arg or the p.Glu96Lys constructs, in similar levels as with the previously described p.Arg30Trp, indicating a dominant interference for these variants (**Figure 2B**). On the other hand, p.Thr117Pro and p.Gln249Pro induced a constitutive expression of GFP in the absence of TCR stimulation, even resulting in higher activation of NF-κB than the one exerted by the reported p.Gly123Ser GOF variant (**Figure 2A**). Although the p.Arg818Gln variant construct showed a slight GOF effect (**Figure 2A**), it did not behave statistically different than control cells (WT/WT) when co-transfected with the WT construct (heterozygous state) and stimulated with anti-CD3/CD28 (**Figure 2B**). Similar results were observed in a NF-κB luciferase reporter assay, where HEK293T cells or Jurkat T cells were transfected with the constructs carrying these variants (**Figure 2C**). CARD11 protein expression was similar for all cell lines carrying constructs with the different variants assessed (**Figure 2**).

### 3.3 GOF CARD11 mutants spontaneously aggregated in the cytoplasm

Previous studies have shown that the expression of *CARD11* GOF mutants can result in multimeric aggregation in cytoplasmic complexes, also called mCADS or signalosomes, in contrast to expression of WT *CARD11* which can be found diffusely spread throughout the cytoplasm (44). This spontaneous formation of the signalosomes has previously been correlated with constitutive activation of NF-κB. In order to further analyze the novel variants p.Thr117Pro and p.Gln249Pro we decided to perform immunofluorescence assays in transfected HEK293T cells to evaluate their effect on subcellular localization. Although HEK293T cells do not express CARD11 endogenously they do express all the factors downstream of CARD11 that are required for NF-κB activation (45).

As expected, cells bearing WT CARD11 produced a diffuse cytoplasmic expression pattern upon staining with an anti-FLAG antibody (**Figure 2D**). In contrast, both cell lines expressing the novel p.Thr117Pro and p.Gln249Pro variants and the already described GOF variant p.Gly123Ser exhibited cytoplasmic aggregates. On the contrary, the cells carrying p.Arg30Trp, pThr43Arg, and p.Glu96Lys mutation as well as those with the p.Arg818Gln variant (not shown) presented diffuse expression throughout the cytoplasm being similar to the WT expression pattern (**Figure 2D**).

Overall, novel variants p.Thr117Pro and p.Gln249Pro were considered GOF while the new variants p.Thr43Arg and p.Glu96Lys behaved as LOF by a DN mechanism.

On the other hand, the only variant identified in our cohort present albeit with a relatively low frequency in gnomAD, p.Arg818Gln, was shown to have no significant impact in CARD11 function as tested by assays known to collaborate in defining pathogenicity. Thus, this family was not considered to be affected by a CARD11-associated disease and was excluded from further analysis.

Furthermore, to better understand the impact of CARD11 variants, we analyzed variant segregation in family members and were able to identify 7 affected relatives in Families 2, 7 and 8 (**Figure 1A**), all of them presenting some clinical and/or laboratory involvement. Therefore, 15 patients from 8 unrelated families from our clinical care center are included to further characterize the range of clinical and immunological phenotypes associated with unique heterozygous germline CARD11 variants, interpreted ex vivo as dysfunctional.

### 3.4 Clinical phenotype of patients with CARD11 heterozygous GOF variants (BENTA)

#### 3.4.1 Demographic features

Five patients from 4 unrelated families with a mean age of 11.8 years (range 6-26 years old) carried 4 unique GOF CARD11 variants. Sex distribution was 1 male and 4 females. The mean age of disease onset was 3.5 months of age (range 2 -24 months). Mean delay in diagnosis was 8 years and 3 months (Range 4-17 years). In all cases, chronic lymphoproliferation was the initial manifestation, and in 40% of them it was accompanied with autoimmune cytopenias at onset. Patient 3A was the only patient with failure to thrive. Detailed demographic information is collected in **Table 1**.

**Table 1:** Demographic, clinical features, immune evaluation, treatment, and outcome in BENTA patients.

Patient	1A*	2A	2B*	3A*	4A*
**Sex (F/M)**	M	F	F	F	F
**Genetic Variant**	c.349A>C p.Thr117Pro	c.367G>A p.Gly123Ser	c.367G>A p.Gly123Ser	c.715_732dup p.239_244dup	c.746A>C p.Gln249Pro
**Age of disease onset**	2 months	Childhood	2 years	2 months	1 years
**Initial manifestation**	Evans Syndrome	Generalized lymphadenopathy and splenomegaly	Splenomegaly	Evans Syndrome	Generalized lymphadenopathy and splenomegaly
**Infections**	Lung EBV viremia	Lung	Lung EBV viremia	Lung EBV and HHV6 encephalitis CMV, HHV-6, BK viremias C. albicans Esophagitis CNS toxoplasmosis with chorioretinitis	Lung (*S. neumoniae*)
**Autoimmunity**	Evans Syndrome Nephrotic Syndrome	No	Evans Syndrome	Evans Syndrome Thyroiditis Hepatitis	Evans Syndrome Factor XI antibody
**Inflammation**	GLILD	No	No	Mouth Ulcers	No
**Splenomegaly**	Yes	Yes	Yes	Yes	Yes
**Lymphadenopathies**	Yes	No	Yes	Yes	Yes
**Atopic disease**	Asthma	No	No	No	No
**HLH**	No	No	Yes	No	No
**Age of Immune Evaluation**	3m	21y	2y	3m	5y
**Lymphocytes** **(cells/mm^3^)**	6562 N	3310 N	17573 ↑	4370 N	11770/↑
**CD3/CD4/CD8** **(cells/mm^3^)**	3018/1181/1443	1190/695/460	4040/1757/2284	2530/1420/1005	4237/2236/1650
**Treg (CD4/CD25^+high^/CD127^+low^)**	N.D	N	N	N	N.D
**CD19 (cells/mm^3^)**	2427/mm^3^↑	1886/mm^3^↑	11770/mm^3^↑	559/mm^3^N	6944/mm^3^↑
**CD19/CD27 total**	1,57%↓	1.26%↓	1.78%↓	2.97%↓	1.73% (11y)↓
**CD19/CD27 IgD+**	0,3%↓	0.53%↓	0.82%↓	1.83%↓	0.89% (11y)↓
**CD19/CD27 IgD-**	1,27%↓	0.73%↓	0.96%↓	1.14%↓	0.84% (11y)↓
**IgG (mg/dL)**	1510 ↑	1320 ↑	2980 ↑	1970 mg ↑	1300 ↑
**IgA (mg/dL)**	83 N	67 N	82 N	242 ↑	62 N
**IgM (mg/dL)**	223 ↑	60 N	163 ↑	400 ↑	20 ↓
**IgE (UI/ml)**	<5 N	<5 N	5 N	6 N	9 N
**Positive Autoantibodies**	Coombs test	N.D.	Coombs test	Coombs test, Anti cardiolipins, ATPO, ATGO, ASMA	Coombs test, Anti Factor XI
**Immunoglobulin Therapy, Indication**	Yes, recurrent infections and poor antibody responses	No	Yes, immune modulation	Yes, immune modulation	No
**Immunosuppressive drugs/Indication**	Steroids Rituximab, MMF/ Evans Syndrome Steroid dependent Nephrotic syndrome, GLILD	–	Steroids ATG Ciclosporin/ HLH	Steroids Sirolimus Rituximab/ Evans Syndrome Autoimmune Hepatitis	–
**HSCT**	Yes (MUD)	No	No	No	No
**Outcome and age**	Deceased, 9 years	Alive, 27 years	Deceased, 6 years	Deceased, 6 years	Alive, 12 years
**Cause of death**	Disseminated ADV infection in the context of primary graft failure	–	HLH	Lung and CNS toxoplasmosis	–

(* marks index case. Evans syndrome was defined as simultaneous or sequential development of autoimmune hemolytic anemia (with positive direct Coombs test) and immune thrombocytopenia. Normal values for lymphocyte subsets taken from Shearer WT, et al. Lymphocyte subsets in healthy children from birth through 18 years of age: the Pediatric AIDS Clinical Trials Group P1009 study. J Allergy Clin Immunol. (2003). 112:973–80. doi: 10.1016/j.jaci.2003.07.003, reference ranges for B cell subsets are from healthy Argentinian children developed by our flow cytometry laboratory. Reference ranges for serum immunoglobulin (IgG, IgA, and IgM) and IgG subclass levels in healthy children. Turk J Med Sci. 2019;49 (2):497-505. Published 2019 Apr 18. doi:10.3906/sag-1807-282L: Reference ranges for serum immunoglobulin E (IgE) taken from Martins TB, Bandhauer ME, Bunker AM, Roberts WL, Hill HR. New childhood and adult reference intervals for total IgE. J Allergy Clin Immunol. 2014;133 (2):589-591. doi:10.1016/j.jaci.2013.08.037; ↑: High for +2 SD of the reference values adjusted for age, ↓:Low for -2 SD of the reference values adjusted for age, N: Within +/- 2 SD for reference values adjusted for age, N.D.: Not done).

#### 3.4.2 Infections

Eighty percent of the patients had at least one serious infectious complication. Recurrent respiratory infections were seen in 4/5 patients, but none developed bronchiectasis. Skin infections were not seen in BENTA patients. Regarding Epstein-Barr Virus (EBV) control, patients 1A and 2B had persistent EBV viremia, while patient 3A presented at 2 years of age EBV and Human herpesvirus-6 (HHV-6) encephalitis with positive PCR for EBV and HHV6 in brain tissue with CD8 lymphocyte infiltrates in brain biopsy. The latter patient developed at 6 years of age a severe cerebral toxoplasmosis infection, with positive viral loads in whole blood for cytomegalovirus (CMV) and HHV-6, BK, and Herpes Simplex Virus-1 (HSV-1), but in the context of severe immunosuppression indicated for her hematological autoimmunity. Detailed infectious history of the patients is collected in **Table 1**.

#### 3.4.3 Autoimmunity

Eighty percent of the patients developed autoimmunity over time. Hematological autoimmunity was the most consistent finding, as 4/5 developed Evans Syndrome. Additional autoimmunity included autoimmune hypothyroidism (1/5), autoimmune hepatitis (1/5), neutralizing Factor XI autoantibodies (1/5) and nephrotic syndrome (1/5).

#### 3.4.4 Lymphoproliferation

**Figure 3:**
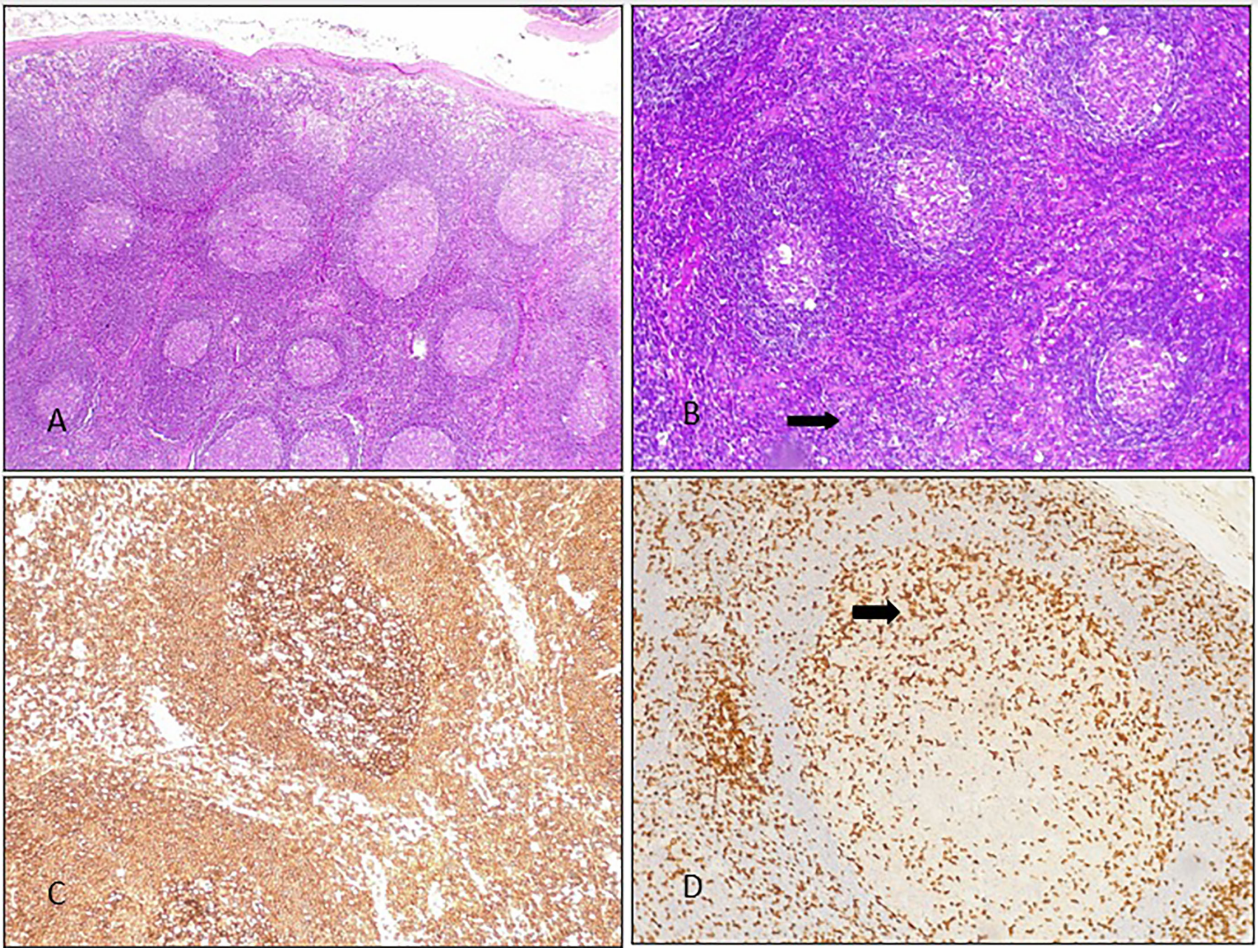
Lymph node histology from BENTA patient 2B: **(A)** several secondary follicles with scant interfollicular tissue. HE 10X. **(B)** Secondary follicles with expanded interfollicular areas were seen (there is some starry sky, arrow). 20X HE. **(C)** CD20: positive lymphocytes in most of the components of the follicle (germinal center and mantle). **(D)** CD3: occasional positive lymphocytes in the germinal center of a large secondary follicle (arrow).

All patients had chronic splenomegaly and 4 of them also had chronic lymphadenopathies (persistent for more than 6 months). Histology from lymph node biopsies showed follicular hyperplasia, in some cases, prominent germinal centers with paracortical expansion of B cells were also described (**Figure 3**). Malignancy and/or infections were ruled out by histology and flow cytometry, and cultures, respectively.

#### 3.4.5 Inflammation: HLH and GLILD

Patient 2B developed recurrent HLH fulfilling 6 criteria, including fever, cytopenias, hepatosplenomegaly, hypofibrinogenemia, hyperferritinemia, hypertriglyceridemia and elevated soluble CD25. She also presented notably generalized nodular skin lesions as well as violet/red erythema around her eyelids. She underwent a skin biopsy that ruled out cutaneous lymphoma but evidenced extensive lymphocyte infiltration. Patient 1A developed Granulomatous Lymphocytic Interstitial Lung Disease (GLILD).

#### 3.4.6 Malignancy

No malignancy was reported in this cohort.

#### 3.4.7 Immunological evaluation

CBC showed lymphocytosis in 2 patients, with no alteration in neutrophils, monocytes or eosinophils. Immune evaluation showed expanded B cells in 4 patients, ranging from 40 to 72% of total lymphocytes. Patient 3A had normal B cells throughout her life. Even though they had expanded or normal B cells, B cell subsets showed decreased CD19^+^/CD27^+^ memory B cells, with diminished CD19^+^/CD27^+^IgD^+^ and CD19^+^/CD27^+^IgD^-^ B cell subsets. Patients 1A and 4A also exhibited expanded transitional B cells (evaluated as CD19^+^/CD27^-^/CD10^+^). Immunoglobulin G was increased in all patients as well. Vaccine responses could not be evaluated in patient 4A since she was receiving immunoglobulin from an early age as immune modulation therapy. Of the remaining 4 patients, patient 2B had normal vaccine responses to protein-based and polysaccharide (pneumococcus) antigens, while patients 1A, 2A and 5A showed diminished responses to some vaccines and/or isohemagglutinins. Regarding T cells, all patients demonstrated normal absolute counts of CD3^+^, CD4^+^, CD8^+^, CD4^+^/CD45RA^+^, CD4^+^/CD45RO^+^. Tregs (CD4^+^/CD25^+high^/CD127^+low^) also showed normal values in the 3 patients evaluated. Likewise, functional evaluation at least by lymphocyte proliferation assay to *phytohemagglutinin* (PHA) was normal in all 4 evaluated patients. We could analyze proliferation to OKT3 only in patient 4A, being defective but with normalization after IL-2 addition. NK cell counts were also within reference values adjusted for age in all patients. See detailed immunological evaluation in **Table 1**.

#### 3.4.8 Treatment and outcome

Immunoglobulin immunomodulatory therapy was used in 3 patients, with patient 1A switching to immunoglobulin replacement therapy as he developed recurrent infections and evidenced poor vaccine responses (**Table 1** and **Supplementary Material**). Two patients had also used Rituximab for Evans syndrome (patient 1A and patient 3A). Other immunosuppressive treatment was needed in 3 patients, 2 for Evans Syndrome (one of them also required it for Steroid Dependent Nephrotic Syndrome) and 1 patient for HLH treatment. Cytopenias in patients 1A and 4A required steroids as first line treatment, Rituximab as second line treatment and we used sirolimus as a steroid sparing agent, which initially showed some improvement, but ultimately failed to control the hematological autoimmunity. HLH was treated with high dose steroids and anti-thymocyte globulin (ATG). No patient required *Pneumocystis jirovecii pneumonia* (PJP) prophylaxis.

Two patients were considered for hematopoietic stem cell transplantation (HSCT) due to severe clinical course. Patient 2B succumbed to HLH before being transplanted. Patient 1A underwent HSCT with a matched unrelated donor due to severe immune dysregulation (GLILD, nephrotic syndrome and refractory autoimmune cytopenias). Conditioning regimen was myeloablative, with Busulfan + Fludarabine +ATG. The patient had primary graft failure. He received a 2nd transplant with the same donor and again suffered a primary graft failure (see **Supplementary Material**).

Three patients died in our cohort. Patient 1A had disseminated adenovirus infection in the context of severe immunosuppression due to primary graft failure following 2 HSCT attempts. Patient 2B had a refractory HLH reactivation with central nervous system involvement. Patient 3A developed cerebral toxoplasmosis (in an immunosuppressed patient because of her refractory hematological autoimmunity). See detailed treatment and outcomes in **Table 1**.

Clinical and immunological profiles alongside functional assays evidencing a GOF effect of the novel *CARD11* variants, confirmed BENTA diagnosis in all 5 patients. Nevertheless, they exhibited extrahematological autoimmune impacts not previously reported and a more severe course for most of them.

### 3.5 Clinical phenotype of patients with *CARD11* Heterozygous LOF variants (CADINS)

#### 3.5.1 Demographic features

Ten patients from 4 unrelated families with a mean age of 7.5 years (range 2-33 years) exhibited 4 unique LOF/DN *CARD11* variants, one per family. Sex distribution was 7 males and 3 females. The mean age of disease onset was 5.9 months of age. In most cases (7/10), atopic dermatitis was the initial clinical symptom, usually severe. Four of the 10 patients had failure to thrive, which coincided with the 4 index cases of the affected families, representing the most symptomatic patients from this cohort. Detailed demographic information is collected in **Table 2**.

**Table 2:** Demographic, clinical features, immune evaluation, treatment and outcome in CADINS patients.

Patient	5A*	6A*	7A	7B*	7C	7D	8A	8B	8C*	8D
**Sex (F/M)**	M	F	M	F	M	F	M	M	M	M
**Genetic Variant**	c.88C>T p.Arg30Trp	c.128C>G p.Thr43Arg	c.286G>A p.Glu96Lys	c.286G>A p.Glu96Lys	c.286G>A p.Glu96Lys	c.286G>A p.Glu96Lys	c.588_589ins42bp p.183_196dup	c.588_589ins42bp p.183_196dup	c.588_589ins42bp p.183_196dup	c.588_589ins42bp p.183_196dup
**Age of disease onset**	9m	6m	Childhood	5m	3m	–	2m	2m	2m	2m
**Initial manifestation**	Atopic Dermatitis	Atopic Dermatitis	Food allergies	Atopic Dermatitis	Bronchial Obstruction	Asymptomatic	Atopic Dermatitis	Atopic Dermatitis	Atopic Dermatitis	Atopic Dermatitis
**Failure to thrive**	Yes	Yes	No	Yes	No	No	No	No	Yes	No
**Infections**	boils and skin abscesses	boils and skin abscesses eczema herpeticum	No	Pneumonia boils Warts	No	*Molluscum contagiosum*	Pneumonia boils and skin abscesses molluscum contagiosum	Pneumonia boils and abscesses Varicella eczema herpeticum molluscum contagiosum Warts	Pneumonia boils and abscesses Warts	boils and abscesses (S. aureus)
**Microorganism isolation**	No	Skin: *S. aureus* Blood: S.Agalactiae SAMS S. pneumoniae	No	No	No	Blood Toxocariasis	Lung: TB	Lung: *S. pneumoniae* Skin: *S. aureus* Blood: *S. pneumoniae* *S. aureus*	Lung: CMV Skin: S. aureus S. epidermidis Blood *S. aureus* *S. epidermidis* *S. maltophilia* *S. pneumoniae* *E. Coli* *C. albicans*	Skin: *S. aureus* Blood: *S. hemoliticus* *S. pneumoniae* CSF: EBV and *S. Pneumoniae*
**Atopic disease and inflammation**	Severe AD Asthma Allergic Rhinitis Eosinophilic Colitis	Severe AD Food Allergies	Mild AD Asthma Allergic Rhinitis Food Allergies	Severe AD Asthma Food Allergies	Mild/Moderate AD Asthma Allergic Rhinitis	No	Severe AD Asthma Allergic Rhinitis	Severe AD Allergic Rhinitis	Severe AD Eosinophilic Colitis	Severe AD
**Distinctive facial features**	No	Yes	No	Yes	No	No	Yes	Yes	Yes	Yes
**Age of Immune Evaluation**	7y	2y	32y	6y	5y	20m	21y	5y	5m	2m
**Eosinophils** **(cells/mm^3^)**	3000 ↑	2550 ↑	1440 ↑	390 N	800 ↑	1270 ↑	740 ↑	198 N	14236 ↑	6320 ↑
**Lymphocytes** **(cells/mm^3^)**	3285	6635	3200	5690	2800	6360	2390	3960	10668 ↑	10820 ↑
**CD3/CD4/CD8** **(cells/mm^3^)**	2628/985/1346	4644/2388/1857	2240/992/1184	4893/2105/2332	2184/952/1092	5090/2863/1972	1720/908/740	2930/1700/1148	8854/5867/2667	6816/3246/3462
**Treg** **(CD4/CD25^+high^/CD127^+low^)**	N	N.D	N.D	N.D	N.D	N.D	N	N	N	N.D
**Lymphocyte proliferation assay to PHA**	N.D.	N.D.	N	↓	N.D.	N.D.	↓	↓	↓	↓
**IgG (mg/dL)**	1320 ↑	718 ↑	912 N	772 N	711 N	569 N	1430 ↑	1620 ↑	364 N	253 ↓
**IgA (mg/dL)**	26 ↓	430 ↑	274 ↑	164 N	148 N	22 ↓	472 ↑	372 ↑	45 N	20 N
**IgM (mg/dL)**	138 N	29 ↓	86 N	86 N	49 ↓	13 ↓	93 N	87 N	61 N	23 ↓
**IgE (UI/mL)**	3190 ↑	4950 ↑	92 N	5330 ↑	1250 ↑	2440 ↑	355 ↑	1800 ↑	7040 ↑	169 ↑
**ImmunoglobulinTherapy, Indication**	No	Yes, low IgM and poor antibody responses	No	No	No	No	Yes, recurrent infections with severe lung damage and poor antibody responses	Yes, recurrent infections and poor antibody responses	Yes,IgG and IgM restriction recurrent infections	Yes, low IgG Skin infections
**Immunosuppressive drugs/Indication**	Steroid Sirolimus Infliximab, Adalimumab/Eosinophilic colitis	Topical steroids/Severe AD	No	Topical steroids Methotrexate/Severe AD	No	No	Systemic and topical steroids/Severe AD	Systemic and topical steroids/Severe AD	Systemic and topical steroids Methotrexate/Severe AD and Eosinophilic Colitis	Systemic and topical steroids/Severe AD
**Outcome and age**	Alive, 22 years	Alive, 4 years	Alive, 33 years	Alive, 9 years	Alive, 6 years	Alive, 2 years	Alive, 27 years	Alive, 21 years	Alive, 6 years	Alive, 4 years

(* marks index case, Normal values for lymphocyte subsets taken from Shearer WT, et al. Lymphocyte subsets in healthy children from birth through 18 years of age: the Pediatric AIDS Clinical Trials Group P1009 study. J Allergy Clin Immunol. (2003). 112:973–80. doi: 10.1016/j.jaci.2003.07.003, Reference ranges for serum immunoglobulin (IgG, IgA, and IgM) and IgG subclass levels in healthy children. Turk J Med Sci. 2019;49 (2):497-505. Published 2019 Apr 18. doi:10.3906/sag-1807-282L; Reference ranges for serum immunoglobulin E (IgE) taken from Martins TB, Bandhauer ME, Bunker AM, Roberts WL, Hill HR. New childhood and adult reference intervals for total IgE. J Allergy Clin Immunol. 2014;133 (2):589-591. doi:10.1016/j.jaci.2013.08.037; ↑: High for +2 SD of the reference values adjusted for age, ↓:Low for -2 SD of the reference values adjusted for age, N: Within +/- 2 SD for reference values adjusted for age, N.D.: Not done).

It is worth noting that 6 patients from 3 unrelated families presented with facial characteristics, including prominent forehead, mild prognathism and increased interalar width of the nose. Retention of multiple primary teeth past the age of typical shedding and high palate were also present in patient 7B.

#### 3.5.2 Infections

Seventy percent of the patients had at least one serious infectious complication. Skin infections were the most frequently found affecting 8/10 patients, being *S. aureus* and *S. epidermidis* the most isolated microorganisms. Cutaneous viral infections were also common (60%), including molluscum contagiosum, *Varicella zoster virus*, HSV-1 infection, and warts. Four patients suffered from lung infections. Among the microorganisms isolated *S. pneumoniae, Mycobacterium tuberculosis*, and CMV could be mentioned but in most cases no isolation was possible. One patient developed bronchiectasis due to recurrent infections. Detailed infectious history of the patients is collected in **Table 2**.

#### 3.5.3 Autoimmunity

Even though some patients presented positive autoantibodies such as antinuclear antibodies (ANA) and anti-smooth muscle antibodies (ASMA), no clinically relevant autoimmunity was documented in this cohort.

#### 3.5.4 Atopy and inflammation

Diverse atopic manifestations were prominent in our cohort. Atopic dermatitis was seen in 9/10 patients in varying degrees of severity, followed by asthma and allergic rhinitis in 5/10, food allergies in 2 of them and environmental allergies in one patient. Patients 5A and 8C developed severe eosinophilic colitis, which was refractory to dietary optimization and required immunosuppression (see Treatment, below). No cases of HLH were documented.

#### 3.5.5 Lymphoproliferation

No chronic lymphoproliferation (defined as splenomegaly or lymphadenopathies for more than 3 months) was observed in this group of patients, only acute enlargement of lymph nodes in the context of skin infections.

#### 3.5.6 Malignancy

No malignancy was reported in our cohort.

#### 3.5.7 Immunological evaluation

Most patients had persistent eosinophilia (80%) (**Table 2**), while no alteration of neutrophils, monocytes and lymphocytes were observed. Likewise, T cell subsets numbers, including CD3^+^, CD4^+^, CD8^+^, CD4^+^/CD45RA^+^, CD4^+^/CD45RO^+^, were within reference values. Additionally, we observed normal values of Treg subset with normal FOXP3 expression in both patients with eosinophilic colitis. Conversely, lymphocyte proliferation assay to PHA was evaluated in 6 patients, with 5 of them (4 belonging to family 8 and patient 7B) showing impairment to PHA. Regarding B cells, all patients presented normal total B cell and B cell subsets counts (CD19^+^/CD27^+^ memory B cells, CD19^+^/CD27^+^IgD^+^ and CD19^+^/CD27^+^IgD^-^ B cells). Serum IgE level was elevated in 90% of the patients, in some cases reaching 30000 IU/ml. Vaccine responses to protein-based and polysaccharide (pneumococcus) antigens could be evaluated in 8 patients (patients 8C and 8D began immunoglobulin replacement therapy from a very young age). Three patients (6A, 8C and 8D) showed diminished responses to some vaccines. Isohemagglutinins were evaluated in 4 patients, being absent in 2 of them. No NK alterations were noted. For detailed immunological evaluation see **Table 2**.

#### 3.5.8 Treatment and outcome

Five patients required immunoglobulin replacement therapy, 4 of whom belong to the same family (family 8), due to recurrent respiratory infections, lung damage and/or impaired antibody-mediated immunity. Five patients are considered to have a combined immunodeficiency, with 3 of them (patients 8B, 8C and 8D) receiving PJP and antifungal prophylaxis. Systemic and topical steroids have been used intermittently in 7 patients, mostly to treat severe atopic dermatitis, and in 2 patients methotrexate was also required. One patient received Sirolimus, Infliximab and Adalimumab due to eosinophilic colitis. Patient 8C was considered for HSCT but as he improved over time, it was ultimately decided against moving forward with the proposed treatment.

From 8 patients presenting severe atopic dermatitis, 4 are now adults. Adult CADINS patients ameliorated or resolved their atopic dermatitis during adolescence and therefore skin infections and the need for medical assistance decreased substantially, with no hospitalizations in the past 5 years. Moreover, 3 of them are not currently receiving topical or systemic treatment for atopic dermatitis. In the pediatric CADINS cohort, two of them are currently only receiving on topical care and one is receiving oral methotrexate, suggesting global dermatological improvement over time. It is worth noting that one patient remained asymptomatic (patient 7D), even though immunological evaluation, e.g., high IgE and eosinophilia placed her undoubtedly closer to CADINS features. Detailed treatment and outcomes are shown in **Table 2**.

## 4 Discussion

Inborn errors of immunity exhibit a wide overlap of clinical phenotypes, including infectious susceptibility, atopy, immune dysregulation, malignancy and in some cases, peculiar facial and dental features. NGS can shorten the time taken to arrive at a diagnosis, but although some identified gene variants provide enough clinical correlation to confirm a disease, other variants require further assessment. In this work, functional assays have aided in evaluating the biological impact of uncertain variants in *CARD11*, enabling us to confirm diagnosis of CARD11-associated diseases in 15 patients from 8 families.

### 4.1 Functional assays for variant validation

Functional assays for the following four *CARD11* variants, novel at the time of their identification p.Thr43Arg, p.Glu96Lys, p.Thr117Pro, and p.Gln249Pro, enabled us to define them as probably pathogenic. We also included another variant, p.Arg818Gln identified in patient 9A with an atypical presentation for a CARD11-associated disease (see Clinical case report in **Supplementary Material**), showing no apparent pathogenic defect in NF-κB activation in the T-cell signaling cascade. Moreover, the presence of this variant in her healthy mother supports our results.

The novel variants showing a LOF/DN behavior p.Thr43Arg and the p.Glu96Lys, are located in the CARD domain of CARD11 where several LOF/DN mutations were already described like that carried by patient 5A. Although the mechanism underlying the DN effect is not fully understood, for some CARD and LATCH LOF/DN variants a disruption in the opening step of CARD11 signaling pathway have been suggested. This in turn could prevent its inducible association with BCL10 and HOIP downstream of TCR activation. Even for some variants, association with cofactors seems to be affected according to CARD11 opening status (46).

As other already reported GOF mutations (e.g. p.Gly123Ser in family 2) p.Thr117Pro variant is found in the LATCH domain of the protein. It has been shown that GOF mutations in the CARD and LATCH domains could exert their effect by disturbing binding to an inhibitory domain, promoting BCL10 association, and inducing BCL10 ubiquitination leading to NF-κB activation (47). It is noteworthy that p.Thr117Pro showed a greater activation of NF-κB *ex vivo* than the reported p.Gly123Ser. Interestingly, in patients with diffuse large B-cell lymphoma the variant p.Thr117Pro had been already reported in somatic state (48). However, no functional analysis was performed at that time. In 2013 another missense variant p.Thr117Ala, generated by random mutation at the same amino acid position in *CARD11* also behaved like a GOF variant (47). Actually, p.Thr117Pro was validated as a GOF variant by using a “cloning-free” approach (49). These collective findings strengthen the idea that mutations at amino acid position 117 of the CARD11 protein could trigger lymphoproliferative phenotypes. Variants p.239_244dup as well as p.Gln249Pro reside in the coiled-coil (CC) domain of the protein where other GOF disease-associated variants, mainly somatic, were already described (https://cancer.sanger.ac.uk/cosmic). Experimental introduction of *CARD11’* CC domain-coding mutants into lymphoma cell lines resulted in constitutive NF-κB activation and enhanced NF-κB activity upon antigen receptor stimulation (42). On the other hand, the variant identified in family 6, p.183_196dup is also located in the CC domain but exerting a hypomorphic effect as other LOF/DN variants. This domain seems to be critical for both CARD11 oligomerization and BCL10-MALT1 interactions (10). Further studies could help improve our understanding in mechanistic properties involved in deploying either GOF or LOF effect.

The functionally irrelevant p.Arg818Gln variant is found in the SH3 domain of CARD11, where up to date no mutation has been reported (50). In fact, between residues 200 to 900, spanning a portion of the CC domain, the PDZ domain, and the SH3 domain no DN mutation has been yet reported (50).

Lastly, immunofluorescence assays showed that all, the well-known GOF p.Gly123Ser mutation and p.Thr117Pro and p.Gln249Pro novel variants caused multimeric aggregation of CARD11 into cytoplasmic complexes in the absence of stimulus. This effect has been previously described as indicative of an active NF-κB signaling. This unique feature of GOF CARD11 mutants could be useful in future clinical management as suggested by Stinson J et al. (44).

### 4.2 Intrafamilial diversity

Familial segregation of new identified variants in *CARD11* is also useful for confirming or dismissing their pathogenicity, as no healthy pediatric carriers have been described until today. For known pathogenic variants, it enables us to establish accurate diagnosis for affected family members. Sanger sequencing allowed to confirm diagnosis in 7 additional affected individuals, mostly clinically reminiscent of a CARD11-associated disease.

In the BENTA cohort, patient 2B suffered HLH with a very severe clinical course with a fatal outcome in early childhood, while her affected mother, patient 2A has only manifested with splenomegaly and few pulmonary infections. On the other hand, in family 7 from the CADINS group, patient 7A suffers from environmental allergies and urticaria, patient 7B presented at 5 months of age with severe atopic dermatitis, patient 7C showed recurrent bronchial obstruction with mild atopic dermatitis while patient 7D remains clinically asymptomatic. Moreover, family 8 had an additional affected boy, patient 8D, born after the initial publication of this family in 2017 (17), presenting a milder phenotype than the 3 other CADINS of his family. Therefore, and even though intrafamilial clinical diversity was already pointed out (23), we found here remarkable differences in phenotypes, ranging from severely compromised to almost asymptomatic individuals. This diversity among those sharing even the same mutation clearly suggests additional factors, like unidentified modifier genes and/or environmental factors, involved in clinical expression. In any case, it is necessary to mention that patient 7D, although asymptomatic yet, shows an immunological phenotype reminiscent to CADINS patients, e.g., high eosinophils and high IgE.

Globally, almost complete penetrance for pathogenic variants in *CARD11* continues to be verified in this report since no pediatric carrier lacking any feature from CADINS or BENTA was found in our cohort.

Otherwise, it is important to note that 3 members of families 6 and 7 from CADINS group manifested atopic diseases, but genetic testing in them revealed a lack of the pathogenic *CARD11* variant found in their families. This highlights the importance of intrafamilial gene analysis, since atopic diseases are rather common occurrences in general population.

### 4.3 New clinical features not previously associated with BENTA

Constitutive canonical NF-κB activity is thought to be responsible for enhanced survival and accumulation of immature and naive B cells in BENTA patients, clinically expressing as splenomegaly, lymphadenopathy, and B cell expansion from an early age as proposed by Snow et al. (13). NF-kB signaling *via* mutant GOF CARD11 may activate pro-survival programs that allow B cells to escape from apoptosis induced by chronic BCR engagement. Furthermore, studies in BENTA patient’s B cells exhibit severely impaired differentiation into plasma cells *in vitro*, despite normal proliferation and enhanced survival after polyclonal stimulation. These differentiation defects also involved low IgG secretion and attenuated induction of key differentiation factors able to lead B cells into plasma cells (51). Indeed, most BENTA patients in our cohort presented with recurrent respiratory infections and alterations in the B cell compartment in number and function, although some of them have improved over time and immunoglobulin replacement therapy was only needed in one patient.

Lymphoproliferation in BENTA is also considered secondary to the enhanced survival of transitional and naive B cells that accumulate in the peripheral lymphoid tissues. Unsurprisingly, all BENTA patients in our cohort had chronic splenomegaly. Spleen, lymph nodes and appendix histology from reported BENTA patients showed florid follicular hyperplasia, containing numerous primary follicles with prominent mantles and marginal zones but few germinal centers (13). Lymph node biopsies from our patients showed similar histological findings, although in some cases, prominent germinal centers with paracortical expansion of B cells were described.

Autoimmune cytopenias are the main reported autoimmune manifestations in BENTA yet involving a reduced number of patients (13, 25, 29). However, in our cohort 80% of patients had severe autoimmune cytopenias, some of them even requiring aggressive management (see Therapeutic Challenges below). We also described extra hematological autoimmunity, such as autoimmune nephrotic syndrome, autoimmune hepatitis, and abnormal coagulation assays due to coagulation factor XI autoantibodies. These autoimmune complications were diagnosed in one patient each, and to the best of our knowledge not previously reported. Moreover, an additional BENTA patient not included in this study, who was followed briefly in our center also developed autoimmune nephrotic syndrome at age 3, demanding as well high dose steroids (unpublished data). Thus, we highlight that hematological as well as extra hematological autoimmunity could be frequent in BENTA patients and can lead to high morbidity.

One patient had normal absolute and relative number of B cells throughout her life, which was surprising considering that B cell lymphocytosis is a hallmark of BENTA. All published patients had expanded B cells with only 3 patients reported by Buchbinder et al. (25). showing a mild B cell lymphocytosis. The latter patients shared the same variant, p.Cys49Tyr, the only reported *CARD11* GOF mutation affecting the CARD domain. The authors speculated that mild B cell lymphocytosis could be related to age dependent bone marrow output, and/or be intrinsically linked to that variant. Our patient showed normal B cells from early life, so age dependent bone marrow output seems unlikely as a possible mechanism. As no other BENTA patient harboring the same variant has been published, we cannot at this time attribute this difference in B cell expansion solely to the variant. Our patient’s variant lies in the LATCH domain as other BENTA variants, excluding a relationship with the protein functional domain. Either way, it is important to highlight that not all BENTA patients present with expanded B cells.

In our BENTA cohort one patient developed GLILD, a term used in the context of interstitial lung disease in IEI. These patients show signs of lymphoproliferative pulmonary disease, including lymphocytic interstitial pneumonia, follicular bronchiolitis, or lymphoid hyperplasia in combination with granulomas (52). The etiology of GLILD is still poorly understood. This pulmonary manifestation is considered as part of a multi-system immune dysregulation, mostly seen in patients with Common Variable Immunodeficiency, but other IEI with immune dysregulation also have the risk of developing GLILD (53). No previous reports of GLILD in BENTA patients have been made. Thus, we could consider BENTA as a dysregulatory IEI that can potentially develop GLILD.

HLH in BENTA was reported at the terminal stage of a patient illness, who did not respond to the treatment (HLH-2004 protocol) and died at the age of 3.5 years (24). Recently, an additional patient was diagnosed with BENTA and HLH, with mild clinical symptoms that did not require a formal HLH treatment, but dexamethasone was taken orally as maintenance therapy (26). In our study one patient had recurrent and refractory HLH). Although we found no evidence of malignancy or an infectious trigger in these episodes, it is worth noting that during her 3rd HLH episode, the patient presented EBV viremia. However, EBV viral loads became negative without specific treatment, suggesting that EBV had a minimal role as a trigger in a patient with many EBV negative episodes. Both BENTA patients previously reported with HLH had a different pathogenic variant than our patient. Thus, although rare, HLH is a potential complication to be aware of, as with other IEI not typically associated with this inflammatory state, such chronic granulomatous disease, severe combined immunodeficiency (54), and STAT1 GOF (55), among others.

T cell anergy is a hallmark of BENTA, as constitutive increased CARD11 activity downstream of the TCR induces a degree of hyporesponsiveness that can be rescued using strong stimuli (56). The two patients from our cohort showing persistent EBV viremia could be related to a T cell defect as well as NK cell dysfunction like previously reported in BENTA patients (13, 20, 55, 56).

### 4.4 New clinical features not previously associated with CADINS

It is well established that CARD11 signals through NF-kB, mTOR and JNK pathways, and that canonical NF-kB activation is impaired in CADINS patients. However, it is not currently known if LOF/DN *CARD11* variants equally affect signaling *via* mTOR and/or JNK, nor which of the dysregulated pathways lead to specific clinical and immunological manifestations in CADINS patients (57). In this context, dysmorphic features have not been so far hardly associated with CADINS patients. To date, prominent forehead, broad nose, poor dentition has been reported in only 2 families, family D in Ma et al., 2017 (17), reported here as family 8, and in six members of a single family (22). In the current study, three additional CADINS individuals from three unrelated families also associated these features. Therefore, three unrelated families in this report presented members with dysmorphic characteristics without sharing *CARD11* mutation. As previously noted (23),, these features present in some but not all affected family members (e.g., family 7) are reminiscent of Hyper IgE Syndrome due to DN mutations in *STAT3* (HIES-STAT3). Defective STAT3-dependent IL11 signaling has been suggested as a potential mechanism for the development of skeletal and connective tissue abnormalities in HIES-STAT3 (58), but it is at present unknown any involvement of CARD11 with skeletal and connective tissues. Recently revised data on HSCT in HIES-STAT3 pointed out its impact on non-immune manifestations such as connective tissue disease (59). At any rate, the growing number of patients identified with these features encourage further investigation.

Several CADINS patients had significant humoral defects with poor specific antibody production leading to increased infections, and requirement of immunoglobulin replacement therapy (a half of our CADINS cohort). It has been suggested that intrinsic defects in B-cell differentiation and/or poor T-cell cooperation could explain this antibody deficiency (23). Indeed, four of the patients receiving immunoglobulin replacement therapy also present defective *in vitro* T cell proliferation assay, consistent with a combined immunodeficiency.

Th2 skewing in lymphocyte differentiation is a prominent feature found in atopic dermatitis (60). Although IFN-gamma expression in CD4^+^ cells has been evaluated in few patients from our cohort, no evidence of decrease was observed (data not shown). Besides, reduced TCR signaling can also lead to Th2 phenotypes (61), and mice models with defective CARD11 signaling also develop atopic dermatitis (62). Atopic dermatitis itself increases the susceptibility to skin infections due to impaired barrier function (63), suppression of cutaneous innate immunity by type 2 inflammation (64), *S. aureus* colonization (65), and cutaneous dysbiosis (66). Nevertheless, most CADINS patients developed a more severe infectious phenotype than classical atopic dermatitis, probably due to the associated B and/or T cell defects.

As seen in our cohort but also from published CADINS patients (50), clinical features can be very broad. Let us cite as an example that CADINS patients can exhibit an IPEX-like presentation (67). Indeed, two patients from our cohort displayed at onset an IPEX-like phenotype, associating atopic dermatitis, eosinophilia, high serum IgE and severe diarrhea, histologically defined as eosinophilic colitis, but keeping normal number of Tregs and lacking endocrine autoimmunity. Thus, *CARD11* should be considered when studying IPEX-like patients, especially in the absence of autoimmunity (although autoimmunity has been reported in up to 20% of CADINS patients (23).

### 4.5 Therapeutic challenges

Consistent with the literature, atopic disease was the common feature present in most CADINS patients, frequently manifesting in childhood as atopic dermatitis (severe in some patients) but also including asthma, allergic rhinitis, food allergies, and even eosinophilic colitis. However, atopy was mild or absent in few patients examined in this report. As previously reported (17), this clinical complication ameliorated in most CADINS patients as they grew older. Our management was in line with other centers, optimizing atopic dermatitis treatment in most patients, and providing immunoglobulin replacement therapy and antibiotic prophylaxis in selected cases. Different immunosuppressive strategies were needed for skin and gut inflammation. Currently, no guidelines exist for the management of CADINS patients. Efforts should be made to develop more precise treatment strategies for better outcomes.

BENTA syndrome has been described in several reports as having a relatively mild course, being most BENTA patients carefully monitored for monoclonal B cell outgrowths and chronic infections with minimal clinical intervention (11). However, in our experience, clinical courses have been far more severe, with fatal outcomes in early life in three of five patients due to refractory autoimmunity, HLH, and/or immunosuppression treatment complications. Given a severe immune dysregulation in our BENTA cohort, different therapeutic options were used, including immunosuppression and HSCT. Despite using different immunosuppressive treatment regimens, three patients could not control their severe immune dysregulation.

HSCT had only been performed in the originally described BENTA patient (12). He first presented with splenomegaly and B cell lymphocytosis in infancy, which persisted into adulthood. In 2006, before the genetic diagnosis was known, he developed B cell chronic lymphocytic leukemia and received HSCT, remaining healthy since 2007 (13). Our sole experience in BENTA and HSCT never achieved primary engraftment and the patient ultimately died. The lack of experience and this bad outcome points to improving our understanding of appropriate conditioning regimens and management. This also highlights the need for better therapeutic options.

## 5 Concluding remarks

The identification of novel *CARD11* mutations in IEI patients with various clinical immune phenotypes and outcomes contribute to better understanding the mechanisms underlying CARD11 pathways in the development of primary immunodeficiencies. It also emphasizes the necessity for available functional studies to establish if rare gene variants associate protein altered activity to guard against the inclusion of individuals carrying variants lacking a functional impact. Given the broad spectrum of clinical phenotypes and the variable severity, CARD11-associated diseases could be more frequent than expected (see overlap with other entities such as common atopic disorders for CADINS and hematologic disorders for BENTA). Thus, it is not surprising that most patients were identified by NGS technologies. Overall, in cases of moderate to severe atopic dermatitis as well as in cases of autoimmunity and lymphoproliferation linked to a B-cell expansion, *CARD11* etiology should be considered. CARD11-associated diseases are a challenging group of disorders from the diagnostic and therapeutic standpoint, and early identification of patients could lead to better outcomes, especially in BENTA cases who may present a more severe progression than expected.

## Data availability statement

The data presented in the study are deposited in the ClinVar repository, accession numbers:VCV001708208.1, VCV001708209.1, SCV002569157.1 , SCV002569154.1, SCV002569156.1, SCV002569153.1, SCV002569151.1 , SCV002569156.1.

## Ethics statement

The studies involving human participants were reviewed and approved by Comite de Etica del Hospital de Pediatria Garrahan. Written informed consent to participate in this study was provided by the participants’ legal guardian/next of kin.

## Author contributions

LU collected the clinical data of the patients and their families, participated in the analysis of the results, created the tables, and contributed substantially to the writing of the manuscript. LE did most of the laboratory experiments, contributed to the analysis and interpretation of the data and in the writing of the manuscript. AP, collected clinical data of patients and families and contributed to the writing of the manuscript. MM and JF participated in the generation of mutants, transfection of HEK293T cells immunoblotting and immunofluorescence assays. EP and VG performed genetic analyzes and revised the manuscript. AB performed immunophenotypical analyzes. MO supervised the study, contributed to the conceptualization of the study, and revised the manuscript. MA supervised the laboratory experiments, contributed to the analysis of the data and collaborate with the writing of the manuscript. SD designed and supervised all the study. All authors contributed to the article and approved the submitted version.

## Funding

This work was supported by grants from the Agencia Nacional de PromociónCientífica y Tecnológica (PICT 2017-2005 and PICT-2017-0352) and Universidad de Buenos Aires (UBACyT 2020), Argentina.

## Acknowledgments

We thank the patients and their families for participating in this research. We also thank Andrew L. Snow for providing CARD11-deficient Jurkat cells (JPM50.6) and pUNO-hCARD11-FLAG construct. We thank María Inés Pérez Millán for the laboratory support.

## Conflict of interest

The authors declare that the research was conducted in the absence of any commercial or financial relationships that could be construed as a potential conflict of interest.

## Publisher’s note

All claims expressed in this article are solely those of the authors and do not necessarily represent those of their affiliated organizations, or those of the publisher, the editors and the reviewers. Any product that may be evaluated in this article, or claim that may be made by its manufacturer, is not guaranteed or endorsed by the publisher.
