# Supplementary material for: Expanding spectrum, intrafamilial diversity, and therapeutic challenges from 15 patients with heterozygous CARD11-associated diseases: A single center experience
Source: Front Immunol. 2022 Nov 3;13:1020927. doi: 10.3389/fimmu.2022.1020927 (PMC9668901; doi:10.3389/fimmu.2022.1020927)

# Supplementary Material

CLINICAL CASE REPORTS

**Patient 1A** is a male boy who presented at 2 months old with Evans Syndrome (autoimmune hemolytic anemia and immune thrombocytopenia) and splenomegaly. At 5 months old he developed a steroid-dependent nephrotic syndrome and at 7 months old, he presented remarkable intraabdominal lymphadenopathies. At 2 years old he presented an episode of polyclonal B cell lymphoproliferation (EBV and CMV negative) that consisted of generalized lymphadenopathy, worsened splenomegaly and a leukocytoclastic vasculitis. At 5 years old, severe respiratory involvement was noted, with shortness of breath, chronic cough, and signs of chronic lung disease such as digital clubbing. He presented numerous bronchiectasis and multiple nodules bilaterally. A lung biopsy allowed for the diagnosis of GLILD, which was treated with rituximab + mycophenolate mofetil, with a partial response. He also presented multiple infections, such as several suppurative acute otitis media and bronchial obstruction.

His immune phenotype showed B cell expansion, with up to 18500 cells/mm3, which were almost entirely naïve. He presented absent IgA and IgE, absent isohaemagglutinins and no response to some T-cell dependent antigens. He was started on SCIg due to the findings mentioned above and continued treatment with sirolimus due to cytopenias that were refractory to MMF.

As he continued to worsen, he underwent Hematopoietic stem cell transplantation (HSCT) 11/12 MUD donor (source: bone marrow), with a low toxicity myeloablative conditioning. He presented primary graft failure and he was subsequently retransplanted two times. He died of respiratory failure due to adenovirus lung disease 10 days later.

**Patients 2A, 2B** belong to the same family, 2B being 2A's daughter. 2A is a female young adult who was studied as a child due to unexplained splenomegaly. She had at age 18 an admission for pneumonia. She did not present any major problems except for a persistent splenomegaly. She had had one child who died at 13 days of life due to severe diarrhea and vomiting. Her immune evaluation revealed B cell expansion with low memory B cells.

**2B** presented at 2 years of age with chronic generalized lymphadenopathy (including abdominal lymphadenopathy) and splenomegaly, weight loss and notable tonsil hypertrophy. At age 6 she developed recurrent HLH. From the immunological point of view, she presented CD19+ lymphocytes of 53%-63 (18000-21000/mm3) on her CBCs persistently. Her B cell profile showed an abnormal distribution with decreased memory B cells. Even though she presented a chronic hypergammaglobulinemia (IgG of 2980 mg/dL, IgM 116 mg/dL) she did not show protective titers against several protein antigens such as HBV, measles, and rubella. She did display serological signs of autoimmunity with positive ASMA and Anti-Actin antibodies. Functional workup for primary HLH did not show any alterations in Perforin or CD107a expression. She only presented a mildly positive EBV PCR that remained constant throughout the HLH process. She fulfilled 6 criteria, which included fever, cytopenias, hepatosplenomegaly, hypofibrinogenemia, hyperferritinemia, hypertriglyceridemia and elevated soluble CD25. She also presented notably generalized nodular skin lesions as well as violet/red erythema around her eyelids. She underwent a skin biopsy that ruled out cutaneous lymphoma but evidenced an extensive lymphocyte infiltration. She was treated with high

dose steroids and anti-thymocyte globulin (ATG) with a good response. Eventually she was discharged but she discontinued follow-up and she died at her province due to multiorgan failure due to reactivation of HLH.

**Patient 3A** is a female girl with early onset multilineage autoimmune cytopenias and splenomegaly at 2 months old that required numerous immunosuppressive treatments. She also presented with autoimmune hepatitis manifested by persistent elevation of transaminases. She had a positive ASMA, with other negative autoantibodies (Anti-Actin and Anti-LKM). Liver biopsy showed a severe chronic hepatitis with a mononuclear infiltrate, presence of plasma cells and eosinophils with ductal damage and proliferation. Even though treated chronically with steroids and sirolimus, transaminases persisted high. Although she had antithyroid antibodies, she didn't present clinically with hypo or hyperthyroidism.

She developed an acute right hemiplegia associated to febrile seizures at 2 years old. The CNS MRI showed a mild communicant hydrocephalus and the presence of left posterior capsular lesion, which was hypointense in T1 and hyperintense in T2 and FLAIR sequences with heterogeneous enhancement with contrast. With these results, a biopsy of the lesions was performed, showing an abundant histiocyte perivascular infiltrate without granulomas, with hemorrhage, microcalcifications and necrosis. Immunohistochemistry showed the presence of CD8+ lymphocyte and a positive PCR for Herpes virus 6 and Epstein-Barr virus, without other microorganisms. She completed treatment with pyrimethamine + sulphadiazine for 8 weeks and ganciclovir, continuing afterwards with valganciclovir prophylaxis. She improved clinically after this treatment but persisted with motor sequelae.

She also suffered significant infections, such as an acquired chorioretinitis by Toxoplasma gondii, impetigo, acute otitis media by Moraxella catarrhalis and low respiratory tract infection by Influenza B. At 5 years old she developed thrombocytopenia that was resistant to multiple immunosuppressive regimes (such as high-dose IVIG, sirolimus, rituximab and high dose steroids), during which she developed multiple infections such as esophageal candidiasis, BK cystitis and herpesvirus 6 reactivation and SNC and lung Toxoplasma gondii infection. She developed respiratory distress with bilateral micronodules and acute encephalitis with new multiple hypodense bilateral lesions and died.

Her immunological workup showed signs of immunodeficiency, particularly in the B cell compartment. Her B cell phenotype showed a normal IgG and IgA with a markedly and persistently elevated IgM in the absence of infection. Strikingly, she presented a normal B cell counts throughout her life, with low B memory cells and high CD21low B cells (45%). Her antibody response was quite diminished for some antigens such as pneumococcus, Hepatitis A, Hepatitis B and varicella, with a good response to rubella and tetanus toxoid. She also presented isohaemagglutinins of 1/1. Her T cell immunological workup showed a normal CD3, CD4 and CD8 counts but CD4/CD45RA was 20%, which is decreased for her age, as well as a gap of 6% for double negative T cells.

**Patient 4A** is a female girl with early chronic lymphoproliferation (splenomegaly and enlarged lymph nodes), who added during infancy recurrent respiratory infections, but has remained relatively healthy with no hospitalizations since adolescence.

**Patient 5A** is an adult male who started follow-up as a child for severe atopic dermatitis with superimposed infections who later developed inflammatory bowel disease. He presented a severe atopic dermatitis (onset at 9 months old) that affected all the body surface, which is refractory to

topical treatment as well as with oral medications such as methotrexate up to today. He has presented multiple boils and skin infections, which haven't required admission. He also had varicella at 4 years old with a benign course.

At 6 years old he developed chronic dysentery with bloody diarrhea and abdominal pain. Intestinal biopsy showed an eosinophilic proctocolitis with severe alteration of the mucosal architecture. This showed mainly colon and rectum involvement, but there was also a mononuclear and polymorphonuclear infiltrate in the ileum.

His symptoms were refractory to steroids, 6-mercaptopurine, sirolimus or the combination of the aforementioned. He started treatment with infliximab at 7 years old with a good response until he was 19 years old, when he was switched to adalimumab.

At 7 years old, persistent bilateral inguinal lymphadenopathy was evidenced. The biopsy showed a reactive paracortical lymphoid hyperplasia with a T, B and plasma cell infiltrate. He was also noted to have persistent severe eosinophilia, for which a bone marrow aspirate was done, ruling out malignancy.

His immune phenotype showed a CBC with persistent severe eosinophilia without cytopenias. He has largely normal lymphocyte subpopulations. Immunoglobulins were unremarkable, except for a sharply elevated IgE of 7900 UI/L. He had an overall good response to T-cell dependent antigens and to polysaccharide antigens. Due to his dermatological and intestinal manifestations, FOXP3 expression and sequencing were done, ruling out IPEX.

**Patient 6A** is a female girl who began follow up for severe atopic eczema when she was 6 months old. She presented several admissions due to extensive impetigo caused by Staphylococcus aureus and 2 episodes of streptococcal bacteremia (one by S. agalactiae and one by S. pneumoniae). She presented an extensive Kaposi varicelliform eruption and a persistent bacteremia by Staphylococcus aureus that also required admission. From the immunological standpoint, she presented CBCs with severe eosinophilia, remarkably elevated IgE and IgA, low IgG and IgM, a normal B cell phenotype but a poor response to polysaccharide antigens (pneumococcal antibodies and isohaemagglutinins) and to some protein antigens such as measles and hepatitis A. She had normal T and NK subsets, normal Th17 cells and a normal DOCK8 expression. She is currently on IVIG supplementation with a good progress after her diagnosis.

**Patient 7A, 7B, 7C, 7D** belong to the same family. The index case, 7B, is a 7-year-old female patient recurrent respiratory infections and atopic dermatitis under the suspicion of Hyper-IgE syndrome. She was born full term/average weight for her age and was otherwise healthy until she was 5 months, presenting with severe and persistent atopic dermatitis which affected the face, the 4 extremities and the trunk, refractory to conventional topical treatments, currently with some improvement with oral methotrexate. She required admission 3 times to treat superinfected eczematous lesions (one of them after varicella). She also presented warts in both hands at 6 years of age which did respond to local treatment. She also refers allergies to dairy and eggs. From the respiratory point of view, she required 9 admissions due to bronchial obstruction and hypoxemia and 1 due to pneumonia. She has up to the moment of this publication a normal CT scan and a respiratory functional test with a mild ventilatory obstruction. She receives preventative treatment with budesonide + formoterol.

She presented low weight and low height for her age. A GH deficiency was ruled out; therefore, her failure to thrive was assumed as multifactorial: multiple infections and prolonged steroid therapy due

to her dermatological involvement. Of note, she has dysmorphic features such as prominent forehead, prognathism and an increased interalar width of the nose. Joint hyperextensibility and a high arched palate were also noted as well as absence of shedding of primary teeth. Immune workup showed severe eosinophilia, no lymphopenia and only a mild CD4/CD8 ratio inversion of 0.9, with otherwise normal T cell and NK subsets, including a Th17 subset of 0.86%. The B cell phenotype showed a mild B cell lymphopenia but with normal B cell subsets. Immunoglobulin levels showed IgG, IgA and IgM within normal range and IgE of 8070 IU/ml (notably elevated).

Antibody responses were globally good against protein antigens such as Varicella, HBV, HAV, Rubella and measles, and low to polysaccharide antigens (S. pneumoniae IgG antibody titers falling after boost with Pneumovax23, and low isohaemagglutinins titers of 1/2).

Her father, 7A, presented food allergies as a child, and later presented sporadic eczema, bronchial obstruction, and mild allergic rhinitis as an adult.

Her younger brother 7B had 2 admissions due to bronchial obstruction without any dermatological problems. He has B cell lymphopenia with preserved memory B cell subsets, a globally normal antibody response and raised IgE of 1250UI/ml.

Notably, her sister 7D is currently asymptomatic, but she has persistent eosinophilia and elevated IgE.

**Patients 8A, 8B, 8C, 8D** belong to the same family and have already been reported, except for 8D, who was born after publication (See Ma et al 2017 for more information).

**8C**, our index patient, was referred at 4 months of age due to a desquamative erythroderma with scarce hair and bilateral inguinal lymphadenopathy to evaluate the possibility of Omenn syndrome, which was ruled out. However, he continued with erythroderma that was a consequence of severe atopic dermatitis, which needed high dose topical and oral steroids. He also developed recurrent warts that were refractory to local treatments. He presented numerous abscesses by Gram positive cocci and numerous episodes of bacteremia by Gram negative bacteria as well as fungi due to the numerous intravenous catheters he needed due to prolonged admissions. In total, he was admitted 15 times for more than 300 days. He also was admitted one time to the ICU due to respiratory distress when a CMV pneumonitis was documented. From a gastrointestinal perspective, he presented a chronic diarrhea. A gut biopsy showed eosinophilic coloproctitis, which was partially responsive to hydrolyzed formula and oral steroids. His overall growth was nonetheless very affected, and he had severe malnutrition and failure to thrive. From a neurological standpoint, he presented afebrile generalized tonic clonic seizures. He had a normal EEG but an opercular temporal dysplasia was evidenced as part of his CNS MRI. These seizures were responsive to clobazam, and he has had a good progression from this perspective ever since. From the hematological perspective, he had a persistent hypereosinophilia. A bone marrow biopsy showed normal hematopoiesis and no leukemic infiltration. He also presented persistent lymphadenopathy; a biopsy was taken, which showed no signs of malignancy but rather follicular atrophy and lymphoplasmacytic depletion.

**8A** is the father of 8C and 8D. He presented severe atopic dermatitis when he was younger, but this improved partially over time. He also had molluscum lesions. He presented a great number of boils and skin abscesses, several episodes of acute otitis media (AOM) and TB pneumonia. He had a chest

CT scan that showed bilateral bronchiectasis and a pneumatocele on the upper right lung due to tuberculosis. He also presented coarse facies, a poor dental status and a noticeable scoliosis.

**8B** is 8A younger brother and 8C and 8D paternal uncle. He presented also severe atopic dermatitis when he was a child, 1 episode of superinfected varicella and several molluscum contagiosum lesions. His eczema is still persistent and severe nowadays.

**8D** is 8C younger brother. He is a male boy who presented a milder phenotype, with moderate atopic dermatitis and staphylococcal infections. He has a normal height and normal weight and has not had as many admissions as patient 8C.

Given this remarkable history, immunological workup was started. A remarkably high IgE was found in all patients. A low IgG and a progressively lower IgM were documented only in patient 8C whereas patient 8A and patient 8B had normal IgG and IgM but remarkably high IgA. Patients 8A and 8B also had a poor response to some protein antigens and polysaccharide antigens. This latter aspect was not evaluated on patients 8C and 8D since they started early on IVIG replacement due to high infection burden. All patients had normal T and NK cell subsets; however, they all failed to proliferate against phytohemagglutinin (PHA) and anti-CD3 with a normal response to other mitogens and antigens. The overall treatment of family 8 has been supportive, based on IVIG/SCIG replacement therapy, antibiotic prophylaxis with co-trimoxazole 5 mg/kg/day PO and topical/oral steroids for their atopic dermatitis.

**Patient 9A** is a female girl who presented at 2 years of age with recurrent HLH, chronic polyarthritis and autoimmune hepatitis. She persisted with disease activity, currently suffering from polyarthritis (elbows, hands, knees, ankles, and feet), and elevated inflammatory biomarkers.

**Supplemental Figure 1**. Custom Inborn Error of Immunity Gene Panel

| | | | | | |
|---|---|---|---|---|---|
| **ACP5** | **DDX58** | **IL22RA1** | **MSN** | **RASGRP1** | **TMEM173** |
| **ADA2** | **DEF6** | **IL23R** | **MVK** | **RBCK1** | **TNFAIP3** |
| **ADAR** | **DNASE1** | **IL24** | **MYD88** | **RELB** | **TNFRSF1A** |
| **AP1S3** | **DNASE1L3** | **IL2RA** | **NEIL3** | **RHOH** | **TNFRSF9** |
| **ARPC1B** | **ERAP1** | **IL2RB** | **NFAT5** | **RIPK1** | **TPP2** |
| **BACH2** | **ERBIN** | **IL36RN** | **NFKB1** | **RNASEH2A** | **TRAP1** |
| **C1QA** | **FAAP24** | **IL6R** | **NFKB2** | **RNASEH2B** | **TREX1** |
| **C1S** | **FADD** | **IL6ST** | **NFKBIA** | **RNASEH2C** | **UNC13D** |
| **C4A** | **FAS** | **IRAK4** | **NHEJ1** | **RNF31** | **USP18** |
| **C4B** | **FASLG** | **IRF1** | **NLRC4** | **RORC** | **WIPF1** |
| **CARD11** | **FBN1** | **IRF8** | **NLRP1** | **RUNX3** | **XIAP** |
| **CARD14** | **FERMT1** | **ISG15** | **NLRP12** | **SAMHD1** | **ZAP70** |
| **CARD9** | **GATA2** | **ITCH** | **NLRP3** | **SH2D1A** | **ZNF341** |
| **CARMIL2** | **IFIH1** | **ITK** | **NOD2** | **SLC29A3** | |
| **CASP10** | **IFNGR1** | **JAK1** | **NRAS** | **SPINK5** | |
| **CD247** | **IFNGR2** | **JAK2** | **OTULIN** | **SPPL2A** | |
| **CD27** | **IKBKB** | **JAK3** | **PEPD** | **STAT1** | |
| **CD3D** | **IKZF1** | **KRAS** | **PIK3CD** | **STAT2** | |
| **CD3E** | **IL10** | **LACC1** | **PIK3CG** | **STAT3** | |
| **CD70** | **IL10RA** | **LCK** | **PIK3R1** | **STK4** | |
| **CD93** | **IL10RB** | **LIG1** | **PLCG2** | **STX11** | |
| **CIB1** | **IL12B** | **LIG4** | **POLA1** | **STXBP2** | |
| **COPA** | **IL12RB1** | **LPIN2** | **PRF1** | **TFRC** | |
| **CORO1A** | **IL12RB2** | **LRBA** | **PRKCD** | **TGFBR1** | |
| **CTLA4** | **IL18BP** | **MAGT1** | **PSTPIP1** | **TGFBR2** | |
| **CTPS1** | **IL1RN** | **MAP3K14** | **PTEN** | **TIRAP** | |
| **CXCR4** | **IL21** | **MCM4** | **RAG1** | **TMC6** | |
| **DCLRE1C** | **IL21R** | **MEFV** | **RAG2** | **TMC8** | |

**Supplemental Figure 2**. Clustal Omega amino acid sequence alignment and conservation between species and CADD scores for *CARD11* single nucleotide variants

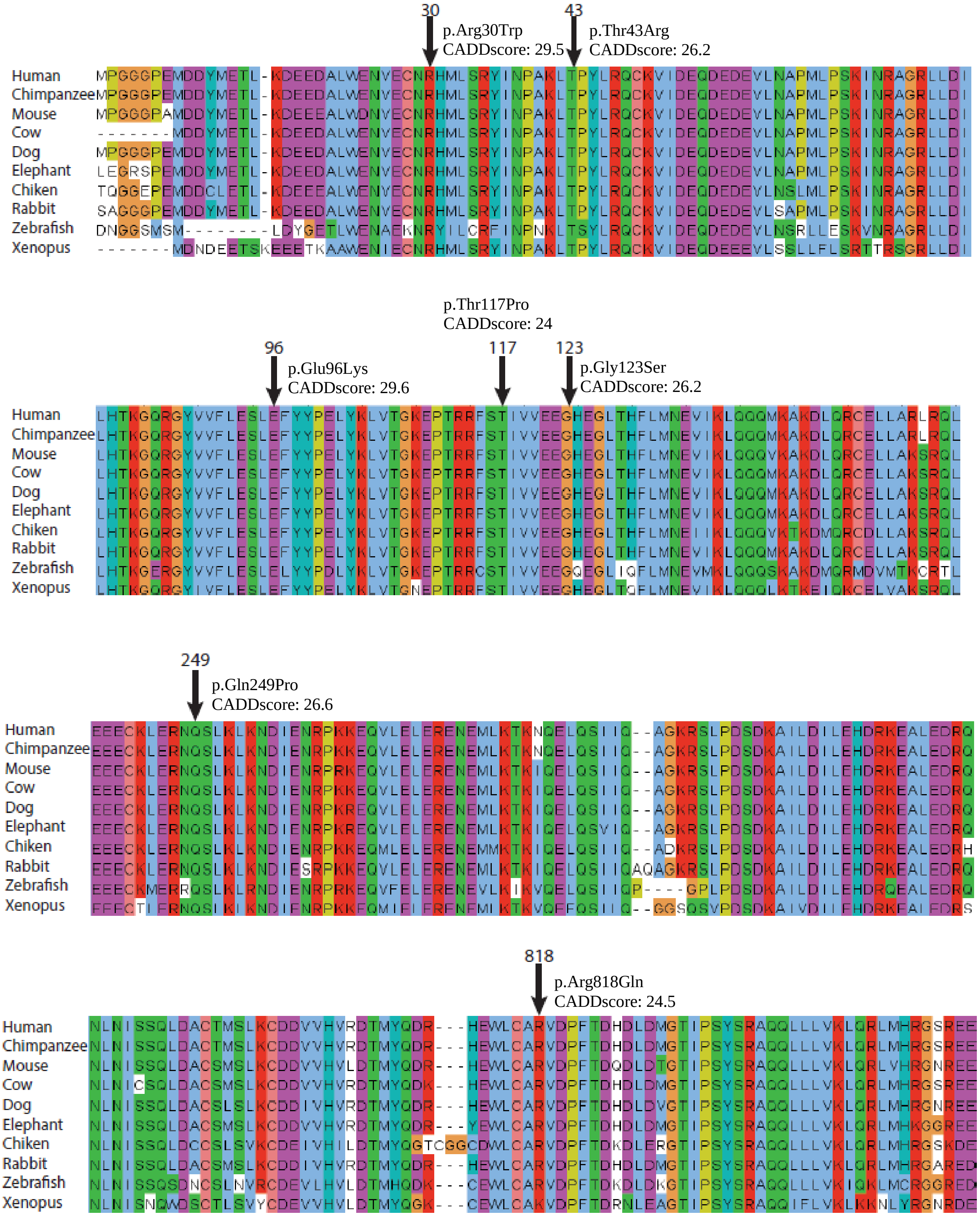

Supplement: Supplementary file 1 [file DataSheet_1.pdf]
